# Single-Molecule Detection of Optical Signals Using DNA-Based Plasmonic Nanostructures

**DOI:** 10.3390/bios15070398

**Published:** 2025-06-20

**Authors:** Renjie Niu, Jintian Shao, Mingnan Wu, Chang Liu, Jie Chao

**Affiliations:** 1 School of Medical Imaging, Xuzhou Medical University, Xuzhou 221004, China; iamrjniu@163.com (R.N.); 202103010308@stu.xzhmu.edu.cn (J.S.); 202102010312@stu.xzhmu.edu.cn (C.L.); 2 The First Clinical Medical College, Xuzhou Medical University, Xuzhou 221004, China; 100002007045@xzhmu.edu.cn; 3 State Key Laboratory for Organic Electronics and Information Displays & Jiangsu Key Laboratory for Biosensors, Institute of Advanced Materials (IAM), Jiangsu National Synergistic Innovation Center for Advanced Materials (SICAM), Nanjing University of Posts and Telecommunications, Nanjing 210023, China

**Keywords:** DNA nanotechnology, single-molecule, plasmonic nanostructures, surface-enhanced Raman spectroscopy, enhanced fluorescence

## Abstract

Single-molecule optical signal detection provides high sensitivity and specificity for the detection of biomolecules and chemical substances, which is of significant importance in fields such as biomedicine, environmental monitoring, and materials science. In recent years, DNA-based plasmonic nanostructures have emerged as powerful tools for achieving single-molecule optical signal detection due to their unique self-assembly properties and excellent optical performance. In particular, DNA origami technology enables the precise construction of metallic nanostructures with specific shapes and functions, which can effectively enhance the interaction between light and matter, thereby significantly increasing signal intensity and detection sensitivity. Furthermore, the programmability of DNA not only simplifies the implementation of single-molecule operations but also allows researchers to design and optimize nanostructures according to specific detection requirements. This review will explore the applications of DNA-based plasmonic nanostructures in single-molecule optical signal detection, including surface-enhanced Raman spectroscopy and enhanced fluorescence for single-molecule signal detection. We will analyze their working principles, advantages, current research progress, and future research directions. By summarizing the work in this field, we hope to provide references and insights for researchers, contributing to the advancement of biomedicine and environmental monitoring.

## 1. Introduction

Single-molecule optical signal detection holds irreplaceable value for exploring the microscopic nature of the material world. As the smallest and relatively stable structural unit, a single molecule exhibits optical properties that often differ significantly from those of bulk systems. These properties provide direct insight into electronic transitions, vibrational modes, and both intra- and intermolecular interactions [1,2,3,4,5,6]. By detecting and analyzing optical signals such as fluorescence or Raman scattering from individual molecules, researchers can investigate molecular kinetics, conformational changes, and quantum behaviors at the molecular level, offering profound implications for understanding microscopic mechanisms and advancing fundamental scientific research [7,8,9,10,11,12,13]. In addition to its scientific significance, single-molecule optical detection offers considerable advantages for practical applications. Compared with traditional detection techniques, it provides markedly enhanced sensitivity and specificity, enabling both qualitative and quantitative analysis of target molecules at ultra-low concentrations or in minuscule quantities. This capability is particularly promising for applications in biomedicine, environmental monitoring, and materials science [14,15,16,17,18].

In conventional single-molecule optical detection, the detection volume is typically large, and molecules diffuse randomly in the bulk. This randomness makes it challenging to accurately localize individual molecules within the detection area, thereby reducing detection efficiency. Moreover, single-molecule signals are inherently weak and often overwhelmed by background noise in complex environments. Researchers typically reduce the detection volume to the femtoliter scale in order to improve the signal-to-noise ratio and suppress background interference. However, at such small volumes—particularly under ultra-low concentrations—molecules take a long time to diffuse into the detection region [13]. To address this limitation, researchers often combine single-molecule detection at low concentrations with techniques such as microfluidics or electrodynamic/electrophoretic trapping, which actively concentrate or transport target molecules into the sensing region. These methods, however, typically require additional instrumentation, making the procedures more complex, labor-intensive, and costly [19,20,21].

DNA nanotechnology offers several advantages for single-molecule detection, particularly in optical platforms employing DNA-based plasmonic nanostructures [22,23,24,25]. DNA can be used to construct highly programmable and customizable nanoscale scaffolds. These scaffolds enable the precise spatial arrangement of metal nanoparticles (e.g., gold or silver), creating localized electromagnetic “hotspots”—regions where confined light–matter interactions dramatically enhance optical fields. Such hotspots are critical for applications like single-molecule sensing or surface-enhanced spectroscopy. DNA enables precise control over the gap size—and thus the electric-field strength at the hotspot—and can position molecules within “high-field, low-background” regions. These capabilities allow DNA-based plasmonic nanostructures to overcome the low signal-to-noise ratios that limit other detection methods, thereby enabling more sensitive optical detection of single molecules. Furthermore, by leveraging the programmability and spatial precision of DNA nanostructures, researchers can anchor probe or target molecules onto predefined DNA scaffolds—particularly within hotspot regions—ensuring optimal spatial alignment with metal nanoparticles. This facilitates single-molecule-level detection. Compared with conventional techniques, DNA-based plasmonic nanostructures offer a simpler and more cost-effective approach to single-molecule optical detection [26,27]. This review summarizes advances in DNA-based plasmonic nanostructures for single-molecule optical detection, including applications in single-molecule surface-enhanced Raman scattering (SERS) and fluorescence enhancement, and discusses current challenges and future opportunities to guide further research in this area.

## 2. DNA Nanostructures

Initially, Professor Nadrian C. Seeman of New York University proposed that cross-branched DNA structures with complementary sticky ends could be spliced to create two- or three-dimensional ordered DNA nanostructures [28]. He further suggested that DNA molecules could serve not only as the genetic material of life but also as a superior material for constructing nanostructures. This marked the beginning of DNA nanotechnology. Owing to its four base components—A, T, C, and G—and the principle of complementary base pairing [29], DNA possesses key advantages such as programmability, sequence specificity, and structural plasticity. DNA nanostructures formed via self-assembly offer ideal platforms for precise assembly, spatial addressability, and target recognition.

**Figure 1:**
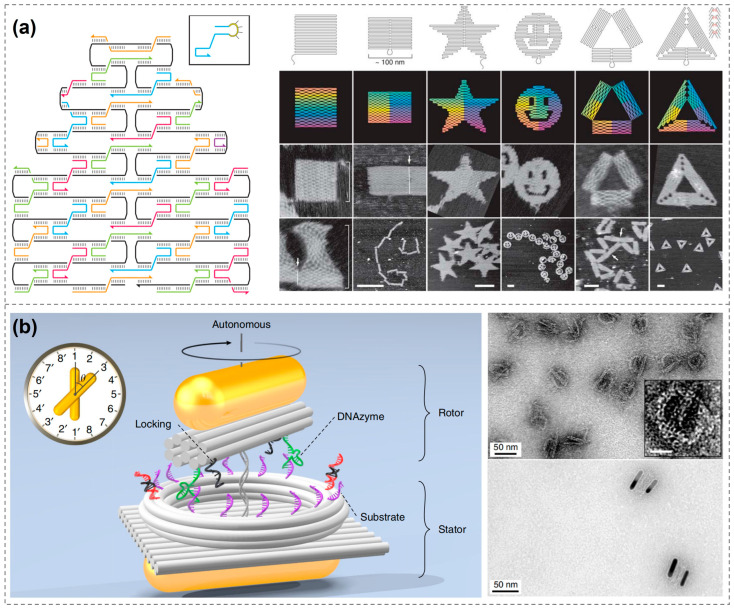
DNA nanostructures. (**a**) Design and construction of DNA origami [30]. Copyright 2006, Springer Nature. (**b**) Clock-shaped DNA origami constructs rotatable chiral AuNR dimers [53]. Copyright 2019, Springer Nature.

The year 2006 marked a pivotal moment in the evolution of DNA nanotechnology. Paul Rothemund of the California Institute of Technology introduced the concept of DNA origami, in which a 7249-base-long circular single-stranded DNA is folded into specific shapes using hundreds of shorter staple strands to fix the structure (Figure 1a) [30]. Over the following decade, DNA origami advanced rapidly. Researchers successfully constructed thousands of multidimensional DNA origami, solidifying the method as a cornerstone of DNA nanotechnology [30,31,32,33,34,35,36,37,38,39,40,41]. DNA origami offers highly programmable templates for organizing molecules and nanoparticles into precise, nanoscale patterns with customizable optical properties, showing great promise in bioanalytical applications [42,43,44,45,46,47,48,49,50,51,52]. For example, Na Liu’s group constructed a clock-shaped DNA origami and used gold nanorods (AuNRs) as the clock hands, creating a rotatable, equipartitioned excitonic nano-“clock” and observing its rotation process via real-time spectroscopy (Figure 1b) [53].

The field of DNA nanostructures continues to develop rapidly, and the research focus is shifting from “What shapes can be made?” to “What functions can be achieved?”. In 2022, the Dietz group constructed a DNA-origami base–platform–rotor device that, when driven by an AC electric field, achieved sustained unidirectional rotation at 250 rpm with measurable torque, showcasing a new platform for programmable nanomotors [54]. In 2023, Seitz et al. employed DNA origami templates to precisely direct the self-assembly of cowpea chlorotic mottle virus (CCMV) and other capsid proteins, enabling programmable control over particle size and morphology, as well as double-layer encapsulation. This strategy enhanced nucleic-acid stability and broadened prospects for vaccine and delivery applications [55]. Zhao et al. recently developed a Cy5/BHQ3-labeled DNA origami platform that is activated by miR-21 to enable fluorescence/photoacoustic dual-modal imaging for early diagnosis of sepsis-associated acute kidney injury (SA-AKI). By simultaneously scavenging reactive oxygen species and enzymatically releasing the antimicrobial peptide LL-37, the platform delivers synergistic therapy that increases the survival rate in disease models to 80% [56]. These breakthroughs underscore the maturation of DNA nanostructures into function-oriented tools and open unprecedented avenues for their deployment in precision medicine, smart materials, molecular machinery, and beyond.

## 3. DNA-Based Single-Molecule SERS Detection

Before the development of complex DNA nanostructures, researchers had already begun utilizing simple DNA double strands to construct various assemblies. Jwa-Min Nam’s group was the first to propose using DNA double strands to enable single-molecule SERS applications. They employed DNA duplexes to link Au nanoparticles (AuNPs), then uniformly grew a Ag shell of controllable thickness on their surfaces, resulting in an Au-Ag core–shell dumbbell-shaped heterodimer. The resulting structures exhibited significantly enhanced Raman signals due to the localized hotspots between nanoparticles, enabling the successful detection of Raman spectra from Cyanine 3 molecules (Cy3, a fluorescent dye molecule widely used in bioimaging). By precisely controlling the thickness of the Ag shells, the structural gap could be finely tuned, resulting in a SERS enhancement factor exceeding 10^12^. They compared the SERS intensity of the Cy3 in Au-Ag core–shell heterodimer, *I_SERS_*, to the intensity *I_SOLUTION_* from the Cy3 solution (33 mM). The enhancement factor was calculated using the recognized formula. Calculating the SERS enhancement factor for plasmonic nanostructures typically uses similar methods. This work established DNA’s foundational role in optimizing SERS hotspots [57].

**Figure 2:**
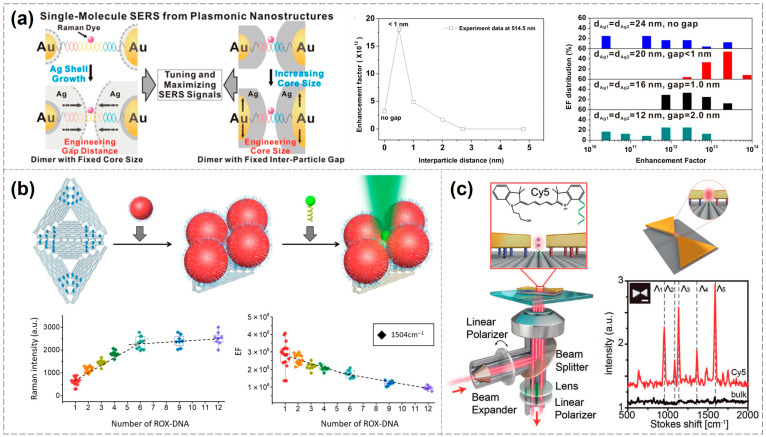
Various configurations of DNA-based plasmonic nanostructures enabling single-molecule SERS detection. (**a**) Influence of the excitation wavelength, as well as the nanogap size, nanoparticle dimensions, and morphology of dumbbell-shaped plasmonic nanostructures, on single-molecule SERS signals [60]. Copyright 2012, American Chemical Society. (**b**) Bowtie structures enabling single-molecule SERS detection [72]. Copyright 2018, Wiley-VCH. (**c**) Quantitative measurement of single-molecule SERS signals using metamolecules [75]. Copyright 2019, The American Association for the Advancement of Science.

This seminal work laid the groundwork for single-molecule SERS detection using DNA nanotechnology. Subsequently, numerous related studies have been published [58,59], and further in-depth investigations have been conducted by Jwa-Min Nam’s group [60,61,62]. For instance, they explored the effects of nanogap size, nanoparticle dimensions, morphology, and excitation wavelength on SERS signals using the nanodumbbells above. When the interparticle gap is less than 1 nm, the resulting hotspots yield exceptionally high Raman enhancement factors—up to 10^13^—with more concentrated and reproducible signal distributions. Conversely, gaps exceeding 1 nm or complete particle contact lead to diminished enhancement and broader signal variability. By increasing the gold core size from approximately 13 nm to 50 nm and varying the thickness of the silver shells, they demonstrated that matching the excitation wavelength to the core-to-shell ratio significantly influences enhancement intensity. Notably, under 514.5 nm laser excitation, the dimers exhibited stronger SERS signals, underscoring the importance of selecting appropriate excitation wavelengths to enhance detection sensitivity. This study highlights that precise tuning of gold–silver core–shell dimer nanogaps via DNA double strands, along with the optimization of nanostructure size, morphology, and excitation wavelength, can achieve highly sensitive and reproducible single-molecule SERS signals. Such advancements are pivotal for the development of high-sensitivity biochemical detection, molecular identification, and related applications using similar nanostructures (Figure 2a) [63].

In addition to dimers, a variety of other nanostructure configurations have been assembled using double-stranded DNA. Jwa-Min Nam’s group, for instance, constructed trimeric gold–silver core–shell nanostructures with varying angles—from acute to linear arrangements—using DNA double strands and inserted single-molecule dye-labeled DNA into each nanogap of approximately 1 nm. By systematically varying the angles of the trimers, they conducted a comparative study of plasmon coupling modes in far-field scattering and near-field enhancement [64]. Beyond the assembly of metallic nanostructures using double-stranded DNA, some researchers have employed double-crossover DNA structures as building blocks to construct two-dimensional nanostructures with highly controllable shapes and rigidity. This strategy allows for precise control of nanoparticle position and spacing. By embedding Cy3 molecules within the DNA strands, they achieved single-molecule SERS signal detection [65].

Although single-molecule SERS detection using plasmonic nanostructures has been achieved with double-stranded DNA, its limited structural design flexibility and restricted spatial precision hinder further development and application. The advent of DNA origami has significantly advanced DNA nanotechnology, offering a more powerful platform for single-molecule detection and driving the field toward greater efficiency and precision [66,67]. In 2013, Bald’s group demonstrated the use of DNA origami to precisely align AuNPs into dimers capable of forming intense electromagnetic hotspots in the interparticle gaps. This approach significantly enhanced SERS sensitivity, enabling the detection of extremely small quantities, even down to individual carboxytetramethylrhodamine (TAMRA) molecules. This work represents the first successful demonstration of single-molecule SERS detection using DNA origami, laying a foundation for the development of highly sensitive nanosensors based on this technique. Moreover, it opens new avenues for customizable and multiplexed single-molecule analysis [68].

In 2014, Liedl and colleagues designed a stacked DNA origami and immobilized AuNPs on both sides, maintaining a spacing of approximately 6 nm. This configuration generated a strong localized electromagnetic field hotspot between the particles. The researchers leveraged the programmability and addressability of DNA origami to precisely position SYBR-Gold (a minor groove-binding fluorescent nucleic acid stain that has a high affinity to double-stranded DNA) within the hotspot region, resulting in a Raman enhancement factor of approximately 3.1 × 10^5^ [69]. In 2016, they refined the DNA origami design by modulating the nanoparticle gap through a photothermal effect, heating-induced shrinkage of the DNA origami, leading to exceptionally strong nanoscale field enhancement [70].

Thacker et al. also developed an AuNP dimer using a three-dimensional DNA origami framework with engineered “grooves” and “ridges”, leaving a controlled gap of around 3–5 nm between the AuNPs. This structure enabled strong field enhancement for the detection of Rhodamine 6G molecules. Both Rhodamine 6G and short DNA oligonucleotide sequences were detected with high sensitivity. Surface enhancement factors ranging from 5 to 7 orders of magnitude were calculated based on the dimer’s orientation within the laser field. This three-dimensional architecture minimizes the interference of the DNA scaffold within the nanoparticle gap [71].

In addition to the commonly used AuNPs, various other nanoparticle morphologies have been employed in the construction of plasmonic nanostructures [72,73]. For instance, Tanwar et. al. assembled a gold nanostar (AuNS) dimer using two rectangular DNA origami and positioned a single Texas Red dye molecule (a sulfonated rhodamine derivative widely used as a red-emitting fluorescent label) at the center of the dimer to achieve single-molecule SERS detection. Compared with AuNP dimers, the “negative curvature” of the AuNS tips enhances plasmonic coupling, making them more effective for single-molecule detection at comparable sizes. The reported enhancement factor reaches up to 2 × 10^10^ when the interparticle spacing is approximately 7 nm and 8 × 10^9^ when the spacing is about 13 nm [74]. Similarly, Baoquan Ding’s group constructed a “bowtie” nanoantenna structure using gold nanoprisms. Owing to the tip effect, this configuration generates significant local electromagnetic field enhancement and enables the detection of Raman signals from single molecules, including general dye molecules as well as alkyne-containing species in the Raman-silent region. The Raman enhancement factor was reported to reach 2.6 × 10^9^ (Figure 2b) [75].

In addition to the classical dimer configuration, various other nanoparticle assemblies can also produce strong electromagnetic field enhancement [76]. Bald’s group employed triangular DNA origami to programmable self-assembled silver nanoparticles of different sizes (10 nm, 20 nm, and 60 nm), forming “nanolenses” for the selective immobilization and detection of individual proteins at hotspots. These nanolenses are self-similar structures composed of sequentially arranged metal nanoparticles of decreasing size within a linear chain. They create localized hotspots between neighboring particles and enable cascaded field enhancement. In essence, nanoparticles of varying sizes act as microscopic lenses, progressively focusing the electromagnetic field toward the smallest particle, where the strongest local field enhancement occurs [77]. Fang et al. utilized a super-origami DNA framework to assemble four 80 nm AuNPs, thereby constructing plasmonic metamolecules capable of generating Fano resonance. At the center of these metamolecules, a specified number of Raman dye molecules were precisely anchored at hotspot regions, enabling quantitative single-molecule SERS signal measurements (Figure 2c) [78].

These studies demonstrate that the SERS enhancement factor is governed by numerous parameters, with each article addressing a distinct subset of these variables. For ease of reference, the factors examined in each study are summarized in Table 1.

As a growing variety of plasmonic nanostructures with diverse configurations continue to be developed for single-molecule SERS detection, they are also finding increasing utility in various applications. For example, in trace or single-molecule detection, amorphous carbon signals can interfere with the detection of target molecules. Bald’s group systematically investigated the mechanisms by which DNA-assembled metal nanostructures generate amorphous carbon during SERS measurements. Using DNA origami, they assembled AuNPs into dimer structures and silver nanoparticles (AgNPs) into nanolens structures at specific positions and performed SERS measurements under varying excitation wavelengths, optical power densities, and substrates. Their results showed that AgNPs are more prone to producing amorphous carbon, and the extent of carbon formation is positively correlated with laser intensity and irradiation duration. Notably, at 785 nm excitation, interactions between AgNPs and silicon substrates led to significantly increased amorphous carbon generation. This study clarifies the underlying mechanisms and contributing factors of amorphous carbon formation and provides valuable guidance for the rational design and application of plasmon-enhanced detection platforms [79].

**Figure 3:**
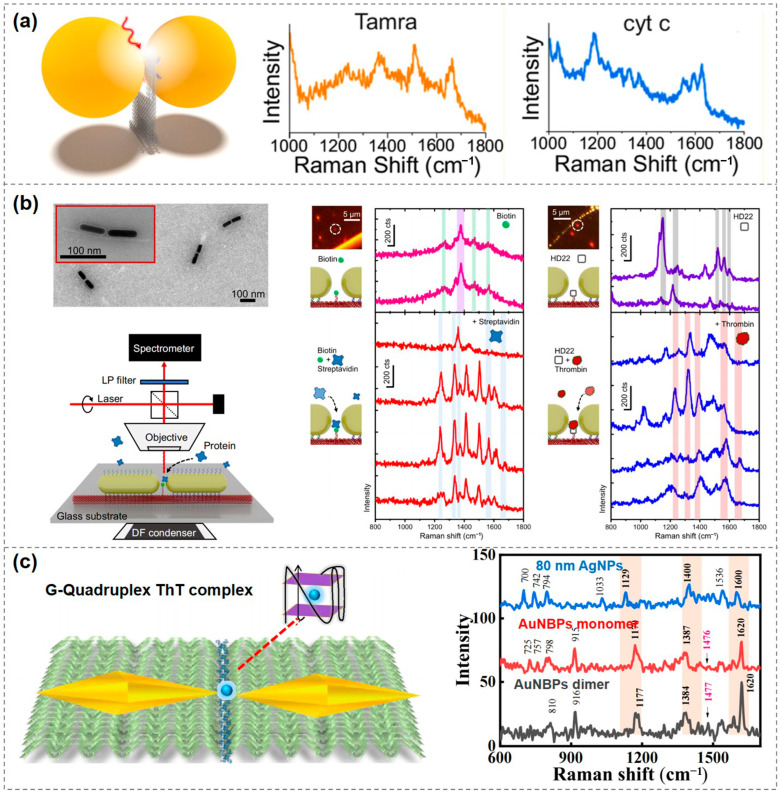
Applications of single-molecule SERS detection using DNA-based plasmonic nanostructures. (**a**) Single-molecule SERS measurements of three dyes, cytochrome c and horseradish peroxidase proteins, enabled by a DNA origami nanofork antenna [26]. Copyright 2021, American Chemical Society. (**b**) AuNR dimers for single-molecule detection of streptavidin and thrombin [79]. Copyright 2023, Springer Nature. (**c**) A plasmonic nanoantenna based on gold bipyramids enabling single-molecule SERS detection of Thioflavin T (ThT) [80]. Copyright 2023, Royal Society of Chemistry.

DNA origami-based plasmonic nanostructures have been widely utilized for the detection of individual protein molecules. In 2021, Tanwar et al. used rectangular DNA origami to assemble Au@Ag nanostars into dimeric structures with varying nanogap sizes, enabling label-free identification of single protein molecules such as thrombin. This approach holds promise for future applications in biomolecular diagnostics and studies of biochemical reactions at the single-molecule level [80]. In the same year, Bald’s group developed a versatile DNA origami nanofork antenna (DONA) by assembling AuNP or AgNP dimers with tunable gap sizes as small as 1.17 nm. This structure enabled SERS signal enhancements of up to 10¹¹, achieving single-molecule SERS detection of three dyes, as well as cytochrome c and horseradish peroxidase proteins (Figure 3a) [26]. In 2022, the same group used the DONA structure to investigate the molecular state of hemin and its spin crossover behavior at the single-molecule level [81]. In 2023, Schuknecht et al. assembled AuNRs into dimers in a tip-to-tip orientation, forming a nanogap capable of accommodating individual protein molecules. Using this configuration, they successfully detected single streptavidin and thrombin molecules and confirmed their characteristic vibrational modes through real-time Raman spectroscopic fingerprinting. This study presents a viable design for near-infrared SERS sensors in single-molecule biomonitoring, with potential applications in single-protein labeling and precision medicine (Figure 3b) [82].

In addition to protein detection, single-molecule SERS can also be applied in other areas. In 2023, Kaur et al. developed a plasmonic nanoantenna based on gold bipyramids that enabled single-molecule SERS detection of Thioflavin T (ThT), a labeling molecule for amyloid protein aggregates. This work lays a solid foundation for future development of highly customizable and scalable SERS platforms aimed at detecting various disease-related biomarkers (Figure 3c) [83].

DNA-based plasmonic nanostructures offer unprecedented design freedom and flexibility for single-molecule SERS detection. Since duplex DNA was first employed to build Au–Ag dumbbell that delivered enhancement factors on the order of 10^12^, researchers have advanced the field to the 10¹³ level by precisely controlling nanogap width, particle size and morphology, excitation wavelength, and laser polarization. At the same time, the detectable analyte range has expanded from small-molecule dyes to proteins, nucleic acids, and even complex biological markers. The advent of DNA origami has further overcome spatial-resolution limits, enabling multidimensional hotspot engineering and detection capabilities.

## 4. DNA-Based Single-Molecule Fluorescence Enhancement Detection

Another rapidly advancing area in single-molecule optical detection is single-molecule fluorescence enhancement. This technique operates on the same principle as single-molecule SERS, utilizing the amplification effects of strong localized electromagnetic fields; however, the focus shifts from Raman scattering to enhancing fluorescence emission intensity. The mechanism behind single-molecule SERS involves the enhancement of Raman signals through interactions with metallic nanostructures, while single-molecule fluorescence enhancement increases emission intensity via localized electromagnetic fields through surface plasmon resonances and modifications of the local density of optical states. The primary advantages of single-molecule fluorescence enhancement include high sensitivity and the ability to detect low-concentration targets with minimal background interference, making it suitable for real-time monitoring of molecular interactions. In contrast, single-molecule SERS provides detailed structural information about the molecules through vibrational modes, offering insights into molecular composition and conformation. However, each technique has its limitations. Single-molecule fluorescence enhancement can suffer from photobleaching, which limits the observation time of fluorescent probes, and its efficiency is highly dependent on the proximity and orientation of the fluorophore relative to the metal substrate. On the other hand, single-molecule SERS tends to require more complex signal processing to extract meaningful data from inherently weak Raman signals, and its sensitivity can be lower compared to fluorescence techniques if not optimized effectively. When combined with precisely programmable nanoscaffolds such as DNA origami, researchers can utilize the spatial control offered by DNA to position metal nanoparticles and fluorescent probes precisely. This enables high-fold fluorescence enhancement of individual molecules at optimized hotspot regions [84,85,86,87,88,89,90,91]. The DNA-based construction of plasmonic nanostructures for achieving single-molecule fluorescence enhancement will be discussed in the following section.

**Figure 4:**
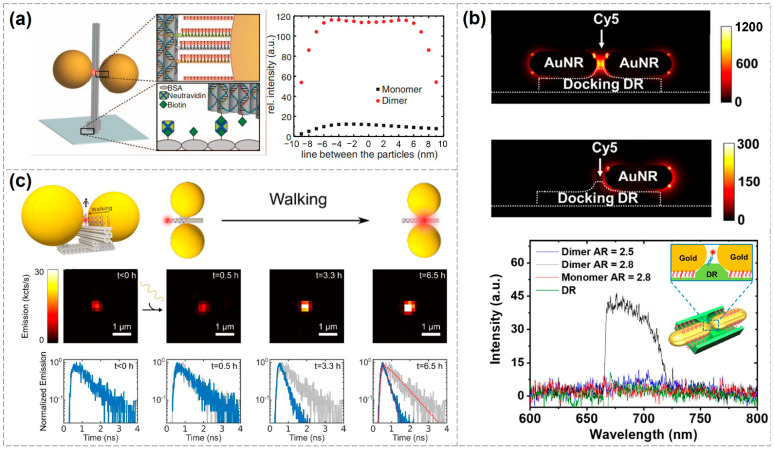
Various configurations of DNA-based plasmonic nanostructures enabling single-molecule fluorescence enhancement detection. (**a**) Detection of single fluorescent molecules using AuNP dimer nanoantennas, achieving a fluorescence enhancement of 117 times [89]. Copyright 2012, American Association for the Advancement of Science. (**b**) Tuning the plasmonic resonance by altering the aspect ratio of AuNRs in the dimers [98]. Copyright 2021, Tsinghua University Press. (**c**) Establishing a single-molecule dye walking mechanism to achieve dynamic monitoring of single-molecule fluorescence signals [99]. The blue solid line represents the change in fluorescence dynamics over time. The decay curve measured before walking (gray solid line) is shown as a reference in the panels obtained at later times. The fits used to extract the fluorescence lifetimes are shown for the last time point (t = 6.5 h) with red dotted lines. Copyright 2019, American Chemical Society.

In 2012, Tinnefeld’s group assembled AuNP dimer nanoantennas using a DNA origami pillar. When the two AuNPs were spaced approximately 23 nm apart, strong plasmonic coupling occurred, forming a hotspot. By placing fluorescent molecules on a pre-designed DNA origami pillar such that they were spatially aligned near the AuNPs, up to a 117-fold fluorescence enhancement was achieved (Figure 4a) [92]. In 2013, the group further refined and extended this method by providing more detailed experimental procedures and numerical simulations to evaluate the effects of nanoparticle size, interparticle gap, and precise dye-to-nanoparticle distance on fluorescence enhancement. They compared fluorescence lifetimes and intensities across three configurations—single particle, two-particle, and no-particle—and found strong agreement between the experimental and simulated results [93]. In the same year, they also achieved precise spatial positioning and orientation of a single AuNP and a single Cy5 dye (Cyanine 5, sulfoindocyanine dye, a far-red-emitting fluorophore with excitation maxima at 649/670 nm) on a DNA origami platform. Using linearly polarized excitation light, they scanned the angular response and observed periodic changes in fluorescence intensity corresponding to the excitation polarization direction. Measurements of single-molecule fluorescence lifetimes revealed that the presence of AuNPs generally shortened dye lifetimes, attributed to AuNP-induced changes in radiative and non-radiative decay rates [94].

In 2015, Tinnefeld’s group achieved significant enhancement of single-molecule fluorescence signals using a 100 nm AuNP dimer with an interparticle gap of 12–17 nm, thereby overcoming the limitations of single-molecule detection at high concentrations. Numerical simulations and experimental verification demonstrated that the local electric field at the antenna hotspot could be enhanced by hundreds to thousands of times when the incident laser polarization was axially aligned with the dimer. This enhancement was particularly pronounced for dye molecules with intrinsically low quantum yields, with the maximum measured fluorescence signal enhancement exceeding 5000-fold. By focusing and amplifying the fluorescence signal of the target molecule, the nanoantenna enabled the resolution of single-molecule fluorescence “blinking” events, even at background dye concentrations as high as 25 μM. This work represents a major advancement in overcoming the long-standing challenge of single-molecule detection under high-concentration conditions [95].

In addition to the classic structure developed by Tinnefeld’s group, other configurations have also been investigated [96,97,98,99]. Chikkaraddy et al. employed DNA origami to immobilize a single Cy5 molecule within the nanogap between a AuNP and a gold mirror. Owing to the highly localized electric field in this nanogap, the single-molecule fluorescence emission was enhanced by several thousand-fold. By controlling the lateral position of the molecule within the nanogap, the authors mapped the spatial distribution of the enhancement factor as a function of distance, thereby achieving a precise mapping of the local field in the nanocavity [100]. Zhao et al. designed a saddle-shaped DNA origami with two grooves that allowed for the precise and controllable end-to-end assembly of AuNRs. The two AuNRs were positioned with a gap of only 2–3 nm, forming an ultrasmall plasmonic nanocavity with a volume of approximately 20 nm^3^, where dye molecules were anchored. By adjusting the aspect ratio of the AuNRs, the plasmonic resonance—and consequently, the emission peak position—could be finely tuned (Figure 4b) [101].

Leveraging the addressability and programmability of DNA origami, it is possible to achieve dynamic monitoring of single-molecule fluorescence. Liu Na’s group employed three-dimensional DNA origami to assemble two 60 nm AuNPs into a plasmonic nanoantenna and implemented a DNAzyme-RNA “walking” mechanism. In this system, individual dye molecules moved step-by-step toward the hotspot region along a predefined track on the DNA origami. By monitoring the dynamics of single-molecule fluorescence, they observed that fluorescence intensity increased while fluorescence lifetime decreased as dye molecules approached the hotspot. This study presents a prototype platform capable of realizing dynamic light–matter interactions at the nanoscale, offering new strategies for designing controllable dynamic plasmonic devices and investigating optical processes at the molecular level (Figure 4c) [102].

**Figure 5:**
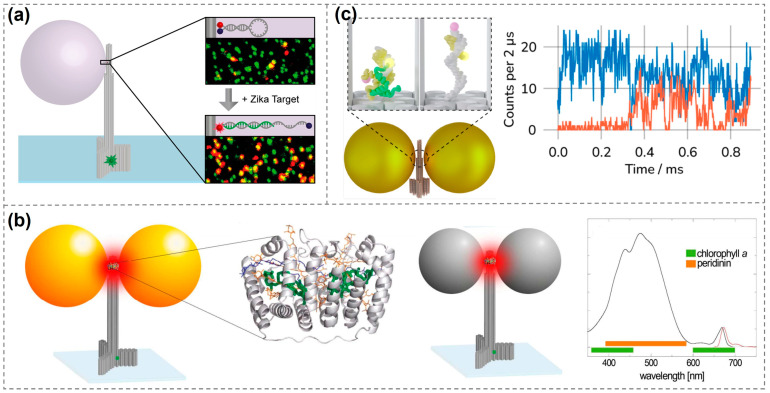
Applications of single-molecule fluorescence enhancement detection using DNA-based plasmonic nanostructures. (**a**) Single-molecule fluorescent detection of specific target sequences, including Zika-specific artificial DNA and RNA [100]. Copyright 2017, American Chemical Society. (**b**) Detection of peridinin–chlorophyll α-protein using metallic nanoantennas [27]. Copyright 2018, American Chemical Society. (**c**) Dynamic observation of single-molecule fluorescence signals for the coupling processes of two proteins and the pairing and dissociation processes of DNA [103]. Exemplary fluorescence time traces (right) showing donor (blue) and acceptor (orange) fluorescence during a hybridization event at 2 μs binning. Copyright 2024, American Chemical Society.

This DNA-based single-molecule fluorescence enhancement approach has also enabled some applications. In 2017, Tinnefeld’s group immobilized fluorescence-quenching hairpins (where energy transfer suppresses emission) with molecular beacons onto DNA origami and positioned them within hotspot regions. When specific Zika virus-related artificial DNA or RNA target sequences were present, the hairpin structures opened and emitted red fluorescence. Combined with plasmonic enhancement, this allowed a single molecule to generate a sufficiently strong fluorescence signal, significantly improving detection sensitivity. This method enables the direct detection of low-abundance target nucleic acids without the need for molecular amplification and demonstrates excellent potential in terms of interference resistance, specificity, and scalability (Figure 5a) [103].

In addition to dye molecules, single-molecule fluorescence detection can also be achieved for weak-emitting porphyrin molecules [104], further expanding the applicability of DNA-based fluorescence enhancement techniques. In 2018, Acuna’s group achieved fluorescence enhancement of over 500-fold by positioning peridinin–chlorophyll α-protein at the hotspot of DNA origami-assembled metal nanoantennas. The study compared enhancement levels under green light (~532 nm) and red light (~640 nm) excitation, revealing that AuNPs enhanced fluorescence more effectively under red light, while AgNPs performed better under green light excitation. By precisely tuning the distance and orientation between the protein and nanoparticles, the researchers systematically analyzed changes in fluorescence intensity and lifetime. These findings demonstrate the feasibility of using DNA origami in combination with metal nanostructures to modulate the optical properties of single molecules, offering a novel strategy for future applications in photonic components and energy conversion based on biological proteins or molecular systems (Figure 5b) [27]. In 2021, Tinnefeld’s group embedded a molecular switch into the hotspot of a plasmonic nanoantenna. The switch contained two antigens that were displaced upon binding with a target antibody, resulting in a measurable fluorescent signal. This approach enabled highly sensitive and specific single-molecule antibody detection. This study employed ATTO 647N (emission wavelength ~ 670 nm) as the reporter fluorophore, paired with the quencher BlackBerry Quencher 650 (BBQ-650) to form a FRET pair. Antibody binding-induced antigen displacement spatially separated the two molecules, restoring ATTO 647N fluorescence. Additionally, ATTO 532 (excitation/emission: 532/553 nm) labeled on the DNA origami structure served as a localization reference dye for spatial identification of the nanosensor in confocal imaging. Notably, the plasmonic coupling between ATTO 647N and the AgNP enabled a 60-fold single-molecule fluorescence enhancement in the NACHOS nanoantenna, a feat unachievable by conventional immunoassay techniques [105]. More recently, they also employed similar structures for the dynamic observation of protein-protein coupling, as well as DNA hybridization and dissociation processes (Figure 5c) [106].

The essence of single-molecule fluorescence enhancement lies in a “close-but-not-too-close” distance engineering: if a fluorophore is placed too near the metal surface, quenching dominates, whereas too large a separation leads to rapid field decay. DNA architectures enable nanometer-accurate positioning of emitters, offering optimal signal amplification and allowing single molecules to be distinguished even against high-background concentrations. As reviewed above, researchers have already demonstrated several proof-of-concept applications. With continued technological advances, single-molecule fluorescence enhancement is expected to evolve toward high-throughput, multiplexed, and even in vivo real-time detection.

## 5. Conclusions and Perspective

In summary, DNA-based plasmonic nanostructures offer high sensitivity, precise controllability, and versatile scalability for single-molecule optical signal detection. The key advantage of DNA nanostructures lies in their ability to provide precise spatial addressability and highly flexible molecular programmability, enabling the ideal alignment of metal nanoparticles and probe molecules at the nanoscale. This results in substantial enhancement of fluorescence or Raman signals and significantly improved signal-to-noise ratios. These techniques have been successfully applied to protein detection, nucleic acid analysis, and the study of complex molecular dynamics, offering novel research avenues and practical opportunities in biomedicine, environmental monitoring, and the development of advanced sensing platforms.

Despite numerous breakthroughs, the field still faces several significant challenges. First, structural reproducibility and large-scale fabrication processes require further optimization to ensure stable and reliable nanoplatforms for practical applications. Thermal expansion, contraction, or folding errors in DNA origami can lead to changes in the configuration of the plasmonic nanostructure, resulting in variations in the electric field intensity of hotspots and shifts in single-molecule positioning, ultimately causing inconsistencies in detection results. To address this issue, it is advisable to use rigid 3D DNA origami, maintain stable experimental temperatures, and employ hydrophilic substrates to reduce the deformation of DNA origami. While these methods may improve the situation, they cannot fundamentally resolve it. We envision that the silicification of the DNA origami could fix the entire structure in the desired shape, thereby ensuring the stability of the results. Second, in real detection environments, conditions are often complex and variable, and effectively controlling the impact of factors such as ionic strength, nonspecific adsorption, and background interference on detection performance remains a significant challenge. In this context, target-irrelevant optical fluctuations could be attenuated by internal-reference calibration, and signal amplification, such as in situ silver deposition, could be employed to enable highly sensitive detection of complex samples. Finally, capturing the transient behaviors of biological systems at the molecular scale and simultaneously detecting multiple components is inherently difficult with this technology alone. Therefore, integration with cross-disciplinary approaches, such as microfluidics [107], is essential to enhance the resolution of dynamic processes.

Looking ahead, with ongoing advancements in DNA origami technology and the growing maturity of nanofabrication processes, DNA-based plasmonic nanostructures are anticipated to enable high-throughput, low-cost, and individually tailored single-molecule detection platforms on a broader scale. These structures are expected to play an irreplaceable role in ultrasensitive biosensing, molecular imaging, and the development of novel quantum optical devices. Integration with emerging fields such as machine learning [108,109] and microfluidics will further expand their application potential, offering new strategies for precision medicine and environmental monitoring. With continued interdisciplinary collaboration and technological integration, DNA nanostructures are poised to remain key drivers of innovation in single-molecule optical detection, laying a solid foundation for future scientific research and industrial applications.

## Figures and Tables

**Table 1:** Studies reported in various articles on factors influencing the SERS enhancement factor.

Factors Influencing the SERS Enhancement Factor	Relevant Articles
Structural gap size	[57,58,59,63,64,70,74]
Structural configuration	[57,64,72,76]
Structural dimensions	[62,63,68]
Structural composition (particle material and morphology)	[57,66,73]
Laser polarization orientation	[57,58,60,61,64,69,75]
Excitation wavelength	[58,62,63,74]
Hot-spot geometry	[62]
Number of dye molecules	[70,78]
Additional influencing factors	Presence or absence of a silica shell [59]; Specific coupling modes [60]; Molecular resonance of the Raman dye [62].
